# Can Universal Health Coverage Be Achieved Through an Unregulated Private Health System? Somalia's Policy Paradox

**DOI:** 10.1002/puh2.70333

**Published:** 2026-08-21

**Authors:** Ilyas Abdullahi Khalif, Aniso Mohamed Abdi, Yusuf Abdullahi Hubow, Ahmed Mohamed Omar, Sharmake Gaiye Bashir, Nuradin Abdullahi Sheikh Rashid, Ahmed Abdinasir Abdulle

**Affiliations:** ^1^ Faculty of Health Sciences and Tropical Medicine Somali National University Mogadishu Somalia; ^2^ Center for Health Research and Innovation Somali National University Mogadishu Somalia; ^3^ Faculty of Health Sciences Salaam University Mogadishu Somalia

**Keywords:** health financing, health system governance, private healthcare sector, somalia, universal health coverage

## Abstract

Universal Health Coverage (UHC) aims to ensure equitable access to quality healthcare without financial hardship. Somalia presents a critical test case for UHC implementation in fragile and conflict‐affected settings due to decades of state collapse, weak governance, and heavy reliance on an unregulated private health sector. This commentary examines Somalia's policy paradox: the coexistence of formal commitment to UHC alongside a predominantly market‐driven, donor‐dependent, and weakly regulated healthcare system. Drawing on evidence from Somalia and comparable low‐income settings, the article argues that unregulated privatization structurally undermines equity, financial protection, and quality of care. High out‐of‐pocket expenditure, fragmented governance, weak regulatory oversight, urban–rural disparities, and donor‐driven financing continue to impede progress toward universal and equitable healthcare access. Although private providers remain indispensable in Somalia's current context, market expansion alone cannot achieve UHC without effective state stewardship, pooled financing mechanisms, and robust regulatory institutions. The commentary concludes that Somalia's pathway toward UHC depends on transitioning from an unregulated commercialized health market toward a regulated mixed health system grounded in equity, accountability, and public‐sector governance.

## 1 Introduction

Universal Health Coverage (UHC) has emerged as a core global health objective, defined as ensuring that all people can access needed, quality health services without suffering financial hardship, with attention to equitable coverage and financial risk protection [1, 2]. This entails not only the availability of essential services, but also their affordability and fair distribution across populations, particularly the poorest and most vulnerable [2, 3].

Somalia, one of the world's most fragile and conflict‐affected states, embodies an extreme stress test for UHC. Decades of civil war, state collapse, and protracted emergencies have devastated public infrastructure, eroded regulatory capacity, and left the country with the lowest UHC index scores globally or regionally [2, 4]. In this vacuum, a predominantly private health market operating within a formally regulated but weakly enforced governance environment expanded, with care financed mainly through out‐of‐pocket (OOP) payments and external aid, and only a minimal share of health spending channeled through government budgets [2, 4]. The result is severe inequities in access, substantial catastrophic expenditure, and widespread unmet need, especially among the poor and rural populations [2, 5].

This commentary interrogates Somalia's policy paradox: a formal commitment to UHC and essential packages of health services coexisting with a health system that is de facto market‐driven, donor‐dependent, and weakly governed [4, 6]. This contradiction raises fundamental questions about equity, affordability, accountability, and state legitimacy in health. The central argument advanced is that achieving UHC through an unregulated private system is structurally implausible in Somalia unless state stewardship, regulatory authority, pooled and pre‐paid financing, and overall health system governance are substantially strengthened [2, 4].

## 2 From State Collapse to Market Medicine

The collapse of Somalia's central government in 1991 triggered the destruction of already modest public health infrastructure: Facilities were vandalized or occupied by militias and displaced populations, thousands of health workers fled or migrated, and the only medical school closed [7, 8]. Prolonged conflict and the emergence of separate administrations in Somaliland, Puntland, and South‐Central Somalia produced a fragmented governance architecture, with parallel ministries and weak national coordination [2, 8]. In this vacuum, state‐led service delivery deteriorated to “virtual non‐existence,” leaving space for rapid expansion of private clinics, pharmacies, laboratories, and informal providers operating largely outside regulatory oversight [9].

Humanitarian crises and recurring disasters further entrenched this model. As public institutions collapsed, international NGOs and UN agencies took over regional hospitals and provided essential services, often through vertical programs and parallel supply and data systems that bypassed government structures [9, 10]. While life‐saving, this aid‐driven architecture fragmented the health system, obscured accountability, and limited the emergence of unified stewardship [10]. Donor dependence, off‐budget financing, and weak federal‐state coordination continue to underpin a patchwork of providers and standards rather than a coherent public system [2, 10].

In this context, healthcare became increasingly commercialized. The system is now “essentially privatized,” concentrated in urban centers, with limited coverage and highly variable quality, in the absence of strong regulatory bodies for facilities, drugs, or professional education [7, 11]. Crucially, this privatized landscape emerged not through deliberate market deregulation but through prolonged institutional collapse, fragmented governance, and limited regulatory implementation, alongside survival‐oriented adaptation by households, health workers, and businesses. This history directly underlies Somalia's current UHC paradox: A de facto market‐led system dominates provision, whereas the state aspires to universal, equitable coverage without yet possessing the regulatory, financing, or stewardship capacity to realign this inherited, unplanned private order toward UHC goals [6, 11].

## 3 Private Sector Dominance and the Limits of Market‐Led Care

Somalia's health system is dominated by a largely unregulated private sector that has become the primary source of care, particularly in urban centers. Evidence from low‐income and fragile settings shows that private providers frequently deliver most basic services, including in Somalia, and have expanded access where public systems are weak or absent [12, 13]. This market‐driven growth has generated employment and filled gaps in service provision, including in tuberculosis care through informal public‐private arrangements [14].

Although evidence consistently documents substantial inequities associated with Somalia's market‐dominant health system, interpretation should acknowledge limitations in measuring health system performance in fragile settings. Estimates of service coverage, OOP expenditure, and catastrophic health spending frequently rely on fragmented administrative systems and household survey data that may be affected by incomplete reporting and recall bias. At the same time, private sector expansion may provide short‐term improvements in service availability where public infrastructure remains weak. In Somalia, private clinics, pharmacies, and diaspora‐supported facilities have contributed to maintaining continuity of selected health services and expanding access in some urban settings. Informal financing and community support mechanisms may also partially alleviate immediate barriers to care for certain households. However, these arrangements remain fragmented, unevenly distributed, and insufficient substitutes for pooled financing, effective regulation, and equitable financial protection required to achieve UHC [2, 12, 14]. However, the structural limitations of this model fundamentally constrain progress toward UHC. Private facilities cluster in better off urban areas, whereas poorer and rural populations face severe geographic and financial barriers, mirroring pro‐rich inequities in access documented in Somalia's UHC index analysis and in broader low‐income country evidence [13, 14].

With approximately 45% of total health expenditure paid OOP and negligible insurance coverage, Somalia's privatized system exposes households to catastrophic spending, impoverishment, and forgone care, directly undermining UHC's financial protection goal [1, 2].

Market incentives also undermine quality and accountability. Reviews of private provision in low‐income and fragile settings highlight suboptimal quality, irrational medicine use, and higher costs relative to public care, particularly where regulation is weak [12, 13]. In Somalia, the absence of an effective regulatory authority has facilitated proliferation of private pharmacies and facilities, fragmented TB partnerships, and inconsistent adherence to clinical standards [2, 14]. Comparative analyses of privatization and financialization show that profit‐oriented models tend to entrench inequalities, segment services by ability to pay, and substitute limited “coverage” for substantive health rights [15].

Consequently, market expansion alone cannot deliver health justice or population‐wide coverage. Although the private sector is indispensable in Somalia's current landscape, its predominantly unregulated, fee‐for‐service configuration risks deepening exclusion and systematically undermining national UHC ambitions unless brought under robust, equity‐oriented governance and financing frameworks.

## 4 Governance Failure and the Regulation Gap

Achieving UHC in Somalia hinges less on the presence of a vibrant private sector than on the existence of coherent governance and robust regulatory institutions. Where stewardship is weak, mixed health systems systematically drift toward inequity, variable quality, and fragmentation rather than convergence on UHC goals [4, 16].

Somalia's health system exemplifies this dynamic: Decades of conflict have produced fragmented governance, limited enforcement of licensing requirements, and historically weak accreditation and inspection systems, resulting in a pluralistic health market that operates under incomplete regulatory implementation rather than complete regulatory absence [4, 6]. Facility accreditation remains rare and uneven, reflecting weak quality assurance and highly variable professional standards across regions [17]. The rapid proliferation of health professions schools without effective accreditation or licensing examinations has further entrenched risks of inadequately trained providers and professional misconduct [17].

Unregulated or weakly governed markets in comparable low‐ and middle‐income countries have generated unsafe clinical practices, irrational antibiotic use, price escalation, and exclusion of high‐risk or poor patients, especially where limited regulatory enforcement and weak insurance oversight expanded private sector influence and increased household costs [16].

Evidence from countries, such as Thailand, Rwanda, and Ethiopia, suggests that private‐sector participation can support UHC only when embedded within strong public stewardship and enforceable regulatory frameworks. Thailand expanded financial protection through tax‐funded purchasing and strategic provider contracting, and Rwanda strengthened access through community‐based health insurance and performance‐based financing, whereas Ethiopia improved service quality through expansion of primary healthcare and stronger facility regulation and accreditation systems. Although Somalia's institutional context differs substantially, these experiences illustrate transferable mechanisms, including phased purchasing reforms, accreditation‐linked provider participation, and selective public contracting rather than unrestricted market expansion [16, 18].

These international experiences also demonstrate that successful UHC reform depends less on adopting a specific financing model and more on sequencing institutional development according to country capacity. Thailand expanded coverage only after sustained public financing and strategic purchasing mechanisms were established; Rwanda combined community‐based financing with local accountability structures and performance incentives; and Ethiopia prioritized primary healthcare expansion before broader insurance ambitions. For Somalia, these examples suggest that immediate priorities should focus on regulatory strengthening, selective contracting of private providers, and progressive expansion of pooled financing rather than attempting rapid nationwide insurance implementation. Transferability therefore lies in governance mechanisms and implementation sequencing rather than direct replication of institutional models [16, 18].

Thus, Somalia's policy paradox is not private‐sector dominance per se, but the attempt to achieve equitable UHC through a market largely unshaped by effective state coordination, regulatory capacity, and accountability mechanisms [6, 16].

## 5 Financial Protection Crisis in Somalia's Health System

Although Somalia has formally endorsed UHC, its predominantly OOP, donor‐dependent, privatized financing model structurally undermines UHC's core goal of financial protection and equitable access.

Somalia's health system is characterized by extreme dependence on OOP payments, which constitute approximately 45% of total health expenditure, far above global and regional averages [1, 5]. Health insurance coverage is negligible (about 3.5%), leaving households exposed to unmediated cost shocks and minimal risk pooling [5]. As a result, roughly a quarter of Somali households face catastrophic health spending, often resorting to borrowing, asset sales, or cutting essential consumption, pushing families deeper into poverty and deterring care‐seeking [2, 5]. This pattern directly contradicts UHC principles that require access “without suffering financial hardship” [1].

The burden of direct payment falls disproportionately on poor households, women, rural communities, and internally displaced persons (IDPs), who already report the highest levels of financial barriers to care and the lowest use of institutional maternity and MNCH services [19]. For these groups, user fees translate into delayed care, untreated conditions, preventable maternal and child deaths, and entrenched social inequities [2, 19].

Financial protection is thus one of Somalia's weakest UHC dimensions: Government spending accounts for only about 5% of health expenditure, whereas donors finance the bulk of public services through fragmented, short‐term humanitarian programs misaligned with national priorities [2]. This aid‐dependent, projectized architecture impedes the development of coherent prepayment and risk‐pooling arrangements, undermines national ownership, and compromises long‑term sustainability [1].

Without substantive reform expanding pooled, compulsory, or subsidized prepayment; regulating private provision; and building robust, equitable financial protection, Somalia's current financing configuration makes the achievement of UHC unattainable.

## 6 Urban Concentration and Rural Health Exclusion

Somalia's predominantly private, market‐led health system is heavily skewed toward urban centers, particularly Mogadishu and other major towns, leaving rural, nomadic, and conflict‐affected populations structurally underserved. Private and NGO facilities cluster where purchasing power and physical security are relatively higher, whereas vast rural and pastoralist areas lack basic services and affordable care [6, 7].

As a result, only about one‐third of Somalis can reach a functional primary healthcare facility within a distance considered geographically accessible under available national and international service access benchmarks (commonly measured as approximately 30 min of travel time or equivalent physical access), and only around 35% have access to essential health services [6, 7].

These spatial and socioeconomic asymmetries translate into profound disparities across the continuum of care. Maternal health indicators are among the worst globally, with maternal mortality around 622–692 per 100,000 live births and extremely low coverage of antenatal care, especially among rural and nomadic women, who face 70%–90% lower odds of adequate ANC than urban women [7]. Institutional delivery and skilled birth attendance remain limited even in major towns, where user fees and indirect costs still exclude poorer women [20]. Pastoralist and remote communities experience chronic shortages of staff, supplies, referral capacity, and transport, further constraining emergency obstetric and child care [21].

Profit‐driven provision reinforces these inequities by concentrating specialist and higher level services, diagnostics, and preventive care in dense, better off urban markets, whereas populations with minimal ability to pay face both geographic and financial barriers, including catastrophic OOP spending and foregone care [22]. These patterned exclusions fundamentally contradict the universality principle of UHC, embedding a tiered system in which access to life‐saving maternal, child, emergency, and preventive services are determined by place of residence and purchasing power rather than need, thereby deepening structural health inequities across Somalia [6, 7].

## 7 Somalia's UHC Policy Paradox

Somalia's formal embrace of UHC coexists with a health system still defined by fragmented, predominantly private and donor‐driven provision, weak regulation, and extreme financial hardship for users. This disjuncture reveals a governance crisis in which UHC is articulated as a technical expansion of services rather than a political project of redistribution, regulation, and accountability.

UHC discourse and planning in Somalia increasingly foreground service coverage through the revised Essential Package of Health Services and the National Transformation Plan, yet implementation failures are repeatedly traced to structural deficits in stewardship, intergovernmental coordination, and regulatory capacity [6, 14]. Despite some gains in coverage for specific interventions, Somalia's UHC index remains among the lowest globally, with deep socio‐economic and educational inequities in access and widespread unmet need [6, 7]. Donor and humanitarian actors have sustained selected programs (e.g., RMNCAH, immunization, and nutrition), often via parallel off‐budget systems that fragment governance, entrench verticalism, and sideline comprehensive financing and institutional reform [9, 10].

The persistence of Somalia's predominantly private and weakly regulated health system also reflects underlying political and economic incentives that shape health sector governance. Urban private providers, privately financed hospitals, pharmaceutical retailers, and externally supported service delivery mechanisms may derive operational advantages from limited regulation and fragmented purchasing arrangements. For households, private provision may offer faster access and perceived reliability where public services remain limited. At the same time, donor‐supported parallel delivery structures can unintentionally reduce incentives for long‐term institutional consolidation. Recognizing these competing interests does not diminish the contribution of non‐state actors but highlights that progress toward UHC depends on aligning incentives across public institutions, private providers, development partners, and communities within an accountable regulatory framework [10, 12, 14].

In this market‐dominant system, care is widely “available” but rarely constitutes UHC: OOP payments account for approximately 45% of total health spending, catastrophic expenditures are common, and financial risk protection remains limited [5, 7].

Evidence from Somalia and other fragile and conflict‐affected states demonstrates that private provision operating under weak regulatory enforcement tends to undermine equity and financial protection unless embedded within strong public purchasing, statutory oversight, and quality assurance mechanisms [13].

Somalia's predicament is therefore less a failure of service expansion than of state capacity and legitimacy. Without decisive investment in public financing, regulatory enforcement (including over proliferating private providers), and accountable federal–state governance, the current model will continue to reproduce exclusion, insecurity, and dependence, rather than deliver UHC as a universal social guarantee.

**FIGURE 1:**
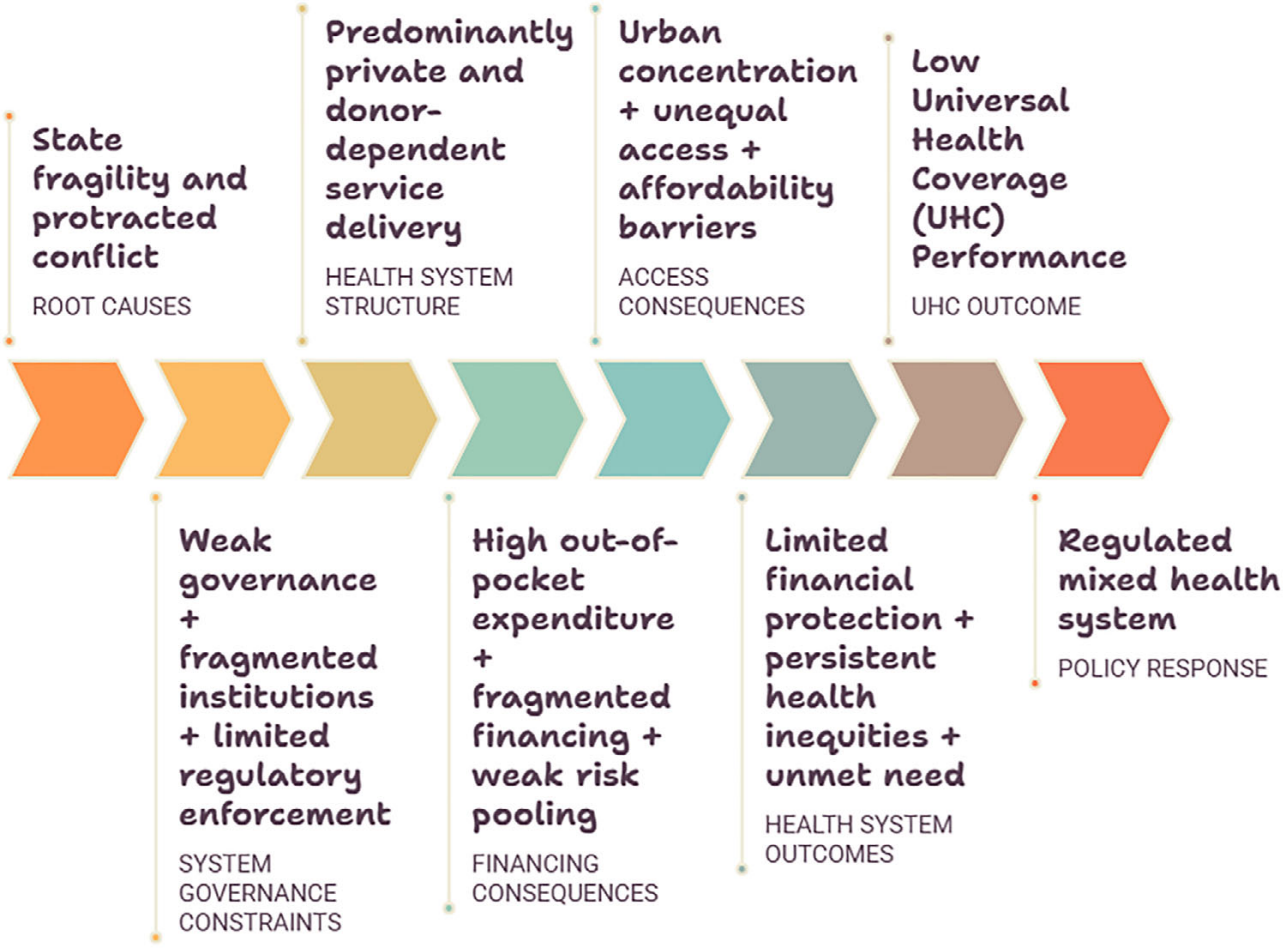
Conceptual framework illustrating Somalia's Universal Health Coverage (UHC) policy paradox and proposed transition toward a regulated mixed health system.

To summarize these interacting structural constraints and proposed reform pathways, Figure 1 presents a conceptual framework illustrating how state fragility, governance limitations, private sector dependence, and fragmented financing collectively shape progress toward UHC in Somalia.

Developed by the authors based on synthesis of evidence presented in this commentary. The framework illustrates how state fragility, governance limitations, private sector dependence, and fragmented financing interact to constrain progress toward UHC in Somalia and highlights proposed policy responses for a more equitable and accountable health system.

The indicators presented in Table 1 are illustrative and intended to operationalize the policy directions proposed in this commentary rather than establish formal national targets. They provide a phased framework linking governance reform, financial protection, and service expansion to measurable progress toward UHC in Somalia.

**TABLE 1:** Illustrative Universal Health Coverage transition priorities for Somalia.

Indicator	Current situation	Proposed target	Timeline	Priority actions
UHC service coverage	Among the lowest globally	Progressive improvement in service coverage	2030–2035	Strengthen PHC and strategic purchasing
Out‐of‐pocket expenditure	Approximately 45% of total health expenditure	<30%	2030–2035	Expand pooled financing and targeted subsidies
Health insurance coverage	Approximately 3.5%	Incremental expansion	2030–2035	Pilot social health protection mechanisms
Access to essential health services	Approximately 35%	≥60%	2030–2035	Rural rehabilitation and service integration
Functional PHC accessibility	Approximately one‐third population coverage	≥60% population coverage	2030–2035	Expand primary healthcare infrastructure
Regulatory oversight	Fragmented and uneven	National licensing and accreditation coverage	2028–2031	Strengthen NHPC and facility regulation

Abbreviations: NHPC, National Health Professionals Council; PHC, primary healthcare; UHC, Universal Health Coverage.

## 8 Reimagining UHC Through a Regulated Mixed System

Somalia's pathway toward UHC requires moving beyond dependence on a predominantly private health market operating within a formally regulated but weakly enforced governance environment toward a regulated mixed system that aligns private sector participation with public goals. Given Somalia's fiscal constraints, fragmented governance structure, and continued donor dependence, progress toward UHC will require phased and context‐specific reforms rather than rapid nationwide financing expansion. Emerging national priorities and evidence from fragile and low‐income settings suggest that meaningful progress is achievable when governance, financing, and primary healthcare are strengthened together [4, 6, 10].

Strengthening governance and regulation should be the immediate priority (2026–2028). The Federal Ministry of Health, Federal Member States, and the National Health Professionals Council (NHPC) should establish a national registry of private facilities and health professionals, strengthen enforcement of licensing requirements, and introduce minimum reporting and quality standards linked to periodic renewal of operating approval. Rather than pursuing broad insurance reforms at this stage, Somalia could pilot selective contracting arrangements in major urban districts whereby accredited private providers deliver components of the Essential Package of Health Services under government oversight. Regulatory reform should also prioritize pharmaceutical oversight through pooled procurement mechanisms, promotion of quality‐assured generic medicines, and transparent pricing approaches to reduce household expenditure and improve medicine availability [5, 6, 18].

In the medium term (2028–2031), reforms should prioritize financial protection and service integration. Expansion of subsidized maternal and child health services, targeted exemptions for poor and internally displaced populations, and selective purchasing of services in underserved districts may strengthen equity while remaining feasible under current fiscal conditions. Strategic purchasing mechanisms, including capitation or blended provider payments linked to licensing compliance, reporting performance, and quality indicators, may improve accountability and cost control [4, 18]. Digital health investments should initially focus on routine health information systems, provider reporting, and referral coordination rather than large‐scale telemedicine deployment, recognizing infrastructure and electricity constraints in remote areas [6].

In the longer term (2031–2035), Somalia should progressively develop social health protection mechanisms through pooled financing approaches supported by domestic revenue mobilization and coordinated donor transition planning [4]. Regulatory institutions should evolve beyond licensing toward facility accreditation, quality monitoring, and performance‐based purchasing [17, 18]. Strengthening primary healthcare through rehabilitation of rural facilities, expansion of female community health workers, and integrated referral systems should remain central to reducing geographic inequities [6].

Achieving this transition will require sustained political commitment, realistic sequencing of reforms, and alignment between government institutions, private providers, development partners, and communities. UHC in Somalia should therefore be understood not solely as a financing agenda but as a governance and accountability project that places equity and public stewardship at the center of health system transformation.

The consequences of delayed action toward UHC extend beyond health financing and service delivery alone. For government institutions, continued fragmentation risks weakening legitimacy and reducing public trust in national systems. For private providers, absence of effective regulation may generate short‐term market growth but undermine long‐term quality assurance and accountability. For development partners, continued reliance on parallel delivery mechanisms may limit institutional sustainability and delay transition toward nationally led health governance. For communities, persistent OOP spending and unequal access threaten social protection and deepen existing inequities. More broadly, Somalia's experience highlights challenges shared across fragile and conflict‐affected settings, where expansion of service availability without corresponding investment in governance, regulation, and pooled financing may fail to translate into equitable UHC outcomes.

## 9 Conclusion

Somalia's experience shows that UHC cannot be delivered by an unregulated marketplace of providers. A predominantly private, donor‐dependent system has produced one of the world's lowest UHC indices, marked by pro‐rich service use, high unmet need, and widespread financial hardship, especially among the poorest households. Heavy reliance on OOP payments, with minimal prepayment and pooling, exposes families to catastrophic spending and forces harmful coping strategies, undermining both equity and efficiency.

Evidence from Somalia and other low‐income and fragile settings demonstrates that although private actors are indispensable for service delivery, commercialization without effective regulation tends to concentrate services in urban areas, raise costs, fragment care, and weaken financial protection. UHC is therefore fundamentally a governance and equity undertaking: It requires strategic purchasing, clear regulation, robust public financing, and mechanisms that prioritize those currently left behind.

Somalia's long‐term progress hinges on rebuilding public institutions, strengthening regulatory bodies like the NHPC, expanding pooled, tax‐funded and insurance‐based coverage, and embedding accountability and anti‐corruption safeguards. Sustainable UHC in Somalia ultimately depends on treating healthcare as a public good under accountable state stewardship, not as a commodity left to unregulated market forces.

## Author Contributions

Ilyas Abdullahi Khalif conceptualized this topic. Nuradin Abdullahi Sheikh Rashid and Ahmed Abdinasir Abdulle supervised this project. Yusuf Abdullahi Hubow conducted a literature review. Aniso Mohamed Abdi and Ahmed Mohamed Omar wrote the first draft. Sharmake Gaiye Bashir revised and edited this manuscript. All authors approved the final version of the manuscript.

## Funding

The authors have nothing to report.

## Conflicts of Interest

The authors declare no conflicts of interest.

## Data Availability

Data sharing is not applicable to this article as no datasets were generated or analyzed during the current study.
